# Intelligent Polymers for Multi-Functional Applications: Mechanical and Electrical Aspects

**DOI:** 10.3390/polym15122620

**Published:** 2023-06-08

**Authors:** Mohammad Rauf Sheikhi, Selim Gürgen

**Affiliations:** 1 Key Laboratory of Traffic Safety on Track of Ministry of Education, School of Traffic & Transportation Engineering, Central South University, Changsha 410075, China; 4121999205@stu.xjtu.edu.cn; 2 Joint International Research Laboratory of Key Technology for Rail Traffic Safety, Central South University, Changsha 410075, China; 3 State Key Laboratory for Strength and Vibration of Mechanical Structures, Shaanxi ERC of NDT and Structural Integrity Evaluation, School of Aerospace Engineering, Xi’an Jiaotong University, Xi’an 710049, China; 4 Department of Aeronautical Engineering, Eskişehir Osmangazi University, Eskişehir 26040, Turkey

**Keywords:** intelligent material, non-Newtonian behavior, multi-functionality, shear stiffening, electrical conductivity

## Abstract

In this study, we fabricated an intelligent material, shear stiffening polymer (SSP), and reinforced it with carbon nanotube (CNT) fillers to obtain intelligent mechanical and electrical properties. The SSP was enhanced with multi-functional behavior, such as electrical conductivity and stiffening texture. Various amounts of CNT fillers were distributed in this intelligent polymer up to a loading rate of 3.5 wt%. The mechanical and electrical aspects of the materials were investigated. Regarding the mechanical properties, dynamic mechanical analysis was carried out, as well as conducting shape stability and free-fall tests. Viscoelastic behavior was investigated in the dynamic mechanical analysis, whereas cold-flowing and dynamic stiffening responses were studied in shape stability and free-fall tests, respectively. On the other hand, electrical resistance measurements were carried out to understand the conductive behavior of the polymers of the electrical properties. Based on these results, CNT fillers enhance the elastic nature of the SSP while initiating the stiffening behavior at lower frequencies. Moreover, CNT fillers provide higher shape stability, hindering the cold flow in the material. Lastly, SSP gained an electrically conductive nature from the CNT fillers.

## 1. Introduction

The properties of intelligent materials can be controlled via external stimulations [1]. The controlled properties can be linked to the mechanical, electrical, magnetic, thermal, rheological, physical, or chemical aspects of the material [2]. Non-Newtonian materials are intelligent materials that change their stiffness depending on the loading conditions, thereby providing variable mechanical responses in different cases. For this reason, researchers have made great efforts to adapt these materials to various engineering applications for designing intelligent systems or structures, benefitting from adaptive behavior. In the pioneering studies, shear thickening fluid (STF) has been recommended for various applications, such as protective composites [3,4,5,6], vibration-damping systems [7,8,9,10,11,12], energy-absorbing structures [13,14,15,16], manufacturing operations [17,18,19,20,21], etc. STF is one of the non-Newtonian materials, consisting of colloidal particles in a liquid medium. This fluid has an increased viscosity under loadings, providing a stiffer texture for users. Despite promising results from the STF, there are some stability problems with this intelligent material [22]. Particle settlement and humidity uptake are the most common drawbacks of STF because the fluid viscosity is heavily affected by these phenomena, hindering long-term usage in applications.

To eliminate the aforementioned shortcomings of STF, a gel-like polymer, namely, shear stiffening polymer (SSP), has been introduced in recent intelligent applications. SSP provides higher stability than fluidic non-Newtonian materials, thereby enabling long-term service life in applications. Tian et al. [23] developed an intelligent gel based on a silicon oil and silicon rubber mixture. The material shows stiffening characteristics under loading. They investigated the mechanical response of this material under various loadings, from static to dynamic conditions. According to the results, the material shows a much stiffer behavior by increasing the silicon oil amount in the composition. Furthermore, the material has a solid-like texture beyond the critical shear strain. Singh et al. [24] fabricated a non-Newtonian material by using silicon oxide, silicon oil, and polyethylene glycol in the mixture. Despite the insoluble nature of silicon oil in polyethylene glycol, a stable mixture is obtained up to a silicon oil rate of 20 wt%. According to the results, the elasticity increases with the silicon oil content in the material because this component acts as a crosslinking agent in the mixture. Silicon oil inclusion is suggested for higher stability in this intelligent material. In another study [25], an SSP was integrated into high-performance textiles. The structures were tested under high-velocity impact conditions to investigate the protective behavior of the SSP. Compared to neat textiles, SSP-integrated fabrics exhibit enhanced energy-absorbing capabilities without compromising structural flexibility. Based on the visual investigations during the impact process, the SSP shows a sudden stiffening, thereby cracking similar to a solid substance upon impact, although the material has cold flowing characteristics at the rest state. Zhao et al. [26] studied the impact behavior of SSP-coated fabrics under high-velocity impacts. The SSP-coated textiles provide about 64% impact force attenuation, whereas the neat textiles show about 27% impact force attenuation. Gürgen et al. [27] integrated SSP into cork-based vibration isolation pads. The SSP shows stiffening under vibrational loading, thereby contributing to the damping behavior in the structures. The damping ratio of the structure is enhanced from 0.025% to 0.145% by including the SSP in the cork pads. Fan et al. [28] used SSP as the core material in the sandwich structures and the structural dynamic properties were investigated. According to the results, the structural stiffness is greatly increased by the SSP inclusion in the composite.

In the present work, we synthesized an SSP and reinforced it with carbon nanotube (CNT) fillers to obtain intelligent properties from mechanical and electrical aspects. Hence, this intelligent material gains multi-functional behavior, such as electrical conductivity and stiffening texture. In the experimental stage, various amounts of CNT fillers (up to 3.5 wt%) were included in the SSP. The polymers were investigated using dynamic mechanical analysis to understand their stiffening behavior. In addition, the polymers were subjected to shape stability and free-fall tests to observe their cold-flowing and dynamic stiffening responses, respectively. Regarding the electrical properties, electrical resistance measurements were carried out to understand the conductive properties. According to the results, the CNT fillers enhance the elastic portion of the SSP while initiating stiffening behavior at lower frequencies. Moreover, CNT fillers provide higher shape stability for the SSP; therefore, cold flowing is significantly hindered in the polymer. On the other hand, SSP gains an electrically conductive nature due to the CNT fillers.

## 2. Experimental Details

### 2.1. Material Fabrication

**Figure 1:**
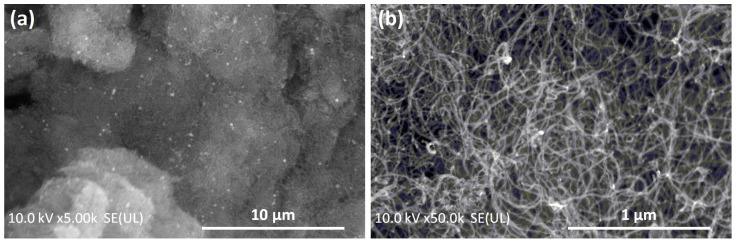
Scanning electron microscopy images of CNT fillers in the SSP at (**a**) 5 k and (**b**) 50 k magnifications.

SSP fabrication was completed using a chemical processing procedure. First, boric acid (from Sigma-Aldrich, Burlington, MA, USA) was heated to 160 °C for 2 h to obtain pyroboric acid. Then, the polymer precursor was obtained by mixing 2 g of pyroboric acid and 15 g of silicone oil (from Basak Kimya, Istanbul, Turkey) in a 1 mL ethanol (from Sigma-Aldrich). The precursor was then reacted at 240 °C for 6 h. After reacting with the precursor, 4 wt% benzoyl peroxide (BPO) (from Sigma-Aldrich) was added to the polymer precursor, and the final mixture was rested at 95 °C for vulcanization for 2 h. To obtain the CNT-reinforced SSPs, various amounts of CNTs (up to 3.5 wt%) were included in the polymer precursor prior to the vulcanization process. The CNT (from Nanografi, Jena, Germany) used in this study was 8–10 nm in diameter and 1–3 µm in length, with a specific surface area of 290 m^2^/g. Figure 1 shows the scanning electron microscopy (Hitachi Regulus 8230) images of the CNT fillers in the SSP. Table 1 lists the design of the specimens used in this study.

**Figure 2:**
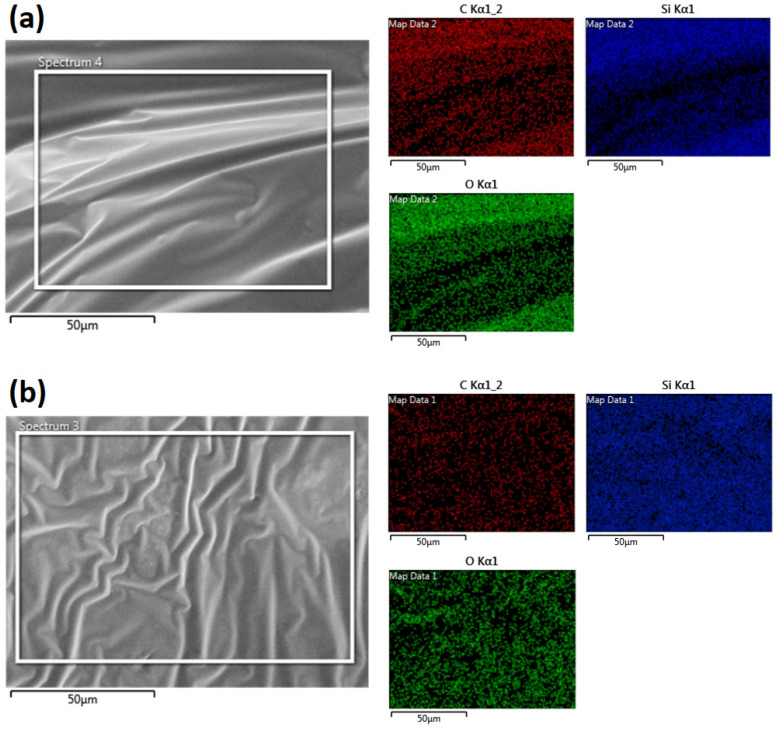
Energy dispersive spectroscopy mapping of the specimens (**a**) SSP and (**b**) SSP/CNT-3.5.

Figure 2 shows the energy dispersive spectroscopy (Hitachi Regulus 8230) mapping results of the pristine SSP and the 3.5 wt% CNT-included SSP. As shown in the images, the distributions of the elements are sufficiently homogenous in the materials. Hence, it is possible to state that the polymers have homogenous microstructures. Dispersing nano-sized fillers in polymer matrices is generally a challenging process [29]. Sharma et al. [30] state that loadings higher than 3–4 wt% of nano-fillers are not suggested due to agglomeration problems in the matrix. It is also noteworthy that CNT fillers are well-dispersed in the SSP matrix despite the agglomeration risk at a filler rate of 3.5 wt%.

### 2.2. Dynamic Mechanical Analyses

The mechanical responses of the polymers were investigated in the rotational mode of oscillatory testing using an Anton Paar MCR 302 system. Standard parallel plates with 25 mm diameter were used for the tests. Shear frequency varied from 0.1 to 100 Hz in the oscillatory tests. Measurements were carried out at a temperature of 20 °C.

### 2.3. Shape Stability and Free-Fall Tests

Spherical balls with a diameter of 40 mm were made from the pristine SSP and 3.5 wt% CNT-included SSP for shape stability and free-fall tests. The specimens were first balled up by hand and then placed into a mold consisting of two hemispherical dies. In the shape stability test, the specimens were rested at room temperature for 1 h. The cold flow behavior of the polymers was recorded using a camera over the resting time. In the free-fall tests, both specimens were released from different heights, and the rebounding heights were recorded to understand the elastic behavior of the polymers.

### 2.4. Electrical Resistance Measurements

The electrical resistance of the polymers was measured using a UT90A multimeter. The probes were spaced 40 mm apart while penetrating 5 mm into the polymers to ensure consistency in the measurements. Moreover, the weight was kept constant at 25 g for all specimens in the measurements.

## 3. Results and Discussion

### 3.1. Mechanical Properties

**Figure 3:**
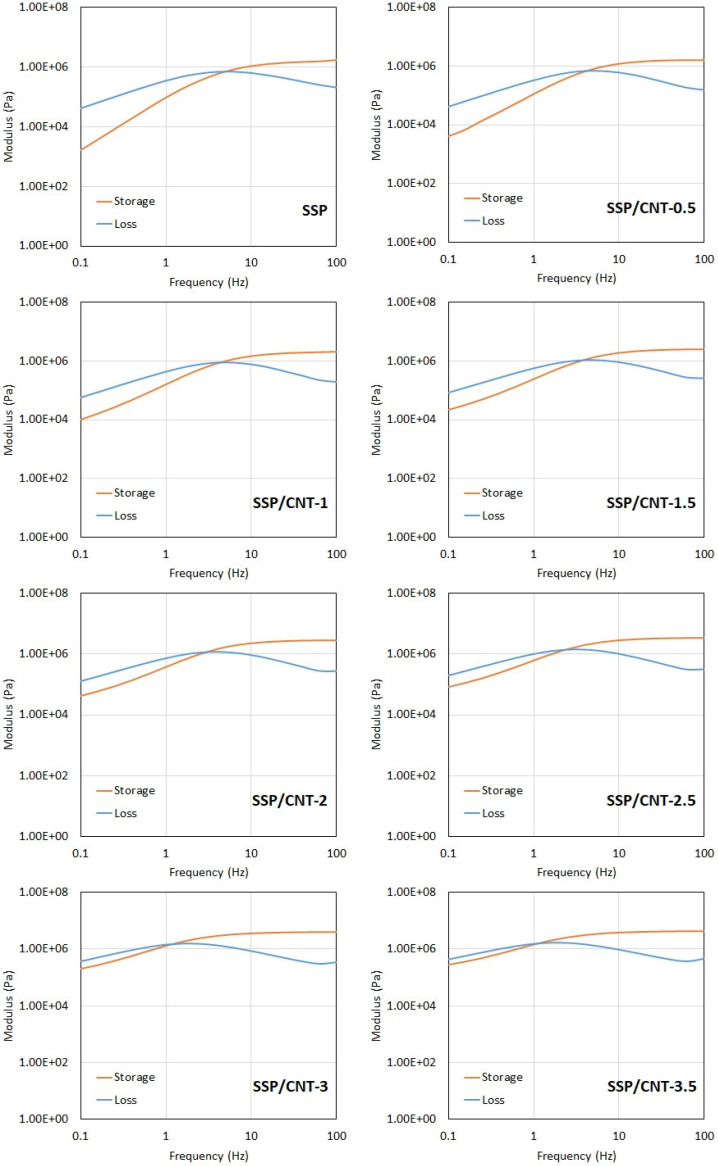
Storage and loss moduli of the specimens.

**Figure 4:**
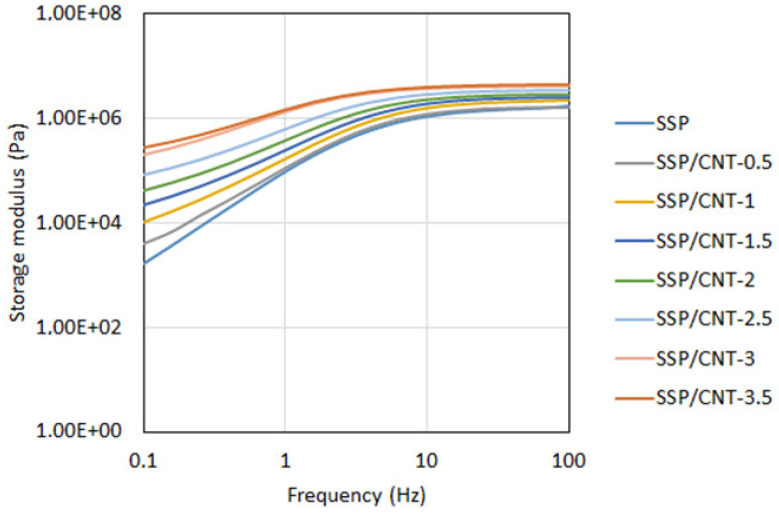
Storage moduli of the specimens.

Figure 3 shows the dynamic mechanical analysis results of the specimens. From these results, it can be seen that there are increasing trends in the storage modulus, which is an indicator of the elastic response in the materials. It should also be noted that the loss modulus shows an increasing trend up to the crossover points in the charts. Beyond these points, a slight reduction is observed in the loss modulus, and the storage modulus predominates over the loss modulus at higher frequencies. This indicates that the polymers exhibit shear stiffening behavior with increasing frequency. In other words, the response of the materials changes from viscous to elastic by increasing the shear in the medium. It can be stated that viscous behavior predominates the material characteristics at lower frequencies; however, the elastic response is pronounced at frequencies higher than 5 Hz for the pristine SSP. The shear stiffening behavior arises from the molecular chains within the polymers [31]. The molecular structure of SSP is formed by long silicon–oxygen (Si-O) bonds. In addition to these fundamental chains, pyroboric acid supplies boron atoms to the medium, thereby forming boron-oxygen (B-O) bonds, which are known as cross bonds between the long Si-O chains [22]. Although the long chains of Si-O bonds can move freely upon loading, the cross B-O bonds restrict the motion of Si-O bonds by tying the long molecular chains to each other. At lower frequencies, the long molecular chains of Si-O bonds find sufficient time for disentanglement, thereby leading to soft characteristics for the material despite the cross bonds. However, loadings accumulate in a short time at higher frequencies, and thus, disentanglement becomes difficult for the long molecular chains within the polymers. In addition to the insufficient disentanglement time, the cross bonds provide an additional constraint for the motion of the long chains. Consequently, the material shows much stiffer characteristics at high-frequency loadings [32,33]. On the other hand, the elastic behavior of the material becomes stronger when more CNT are included in the polymer. One can understand this key feature simply by checking the crossover points with respect to CNT loading. From the results, the crossover point is at about 5 Hz for the pristine SSP, whereas it slightly reduces when more CNT are added to the polymer. Finally, the crossover point is observed at about 1.5 Hz for the SSP with a CNT loading of 3.5 wt%. Another indicator is the upward shift in the storage modulus with increasing CNT content in the polymer, as shown in Figure 4. It can be clearly seen in the chart that the storage modulus becomes stronger as the CNT amount increases in the material, so the elastic response predominates the polymer. This is associated with the mechanical properties of CNT that provide a reinforcing effect for the polymer matrix. As the CNT loading increases in the material, it occupies a larger volume in the matrix, thereby restricting the molecular motions within the polymer and hindering the disentanglement of molecular chains due to the increasing internal friction. Because CNT has higher rigidity and mechanical strength in comparison to the polymer matrix, CNT leads to a pinning effect for the molecular chains by acting as anchor points without showing any deformation on their bodies [31]. Despite significant development in the elastic portion of the polymer, the shear stiffening characteristics are not fully destroyed by CNT additives. Viscous behavior still prevails over relatively low frequencies just before the crossover points.

**Figure 5:**
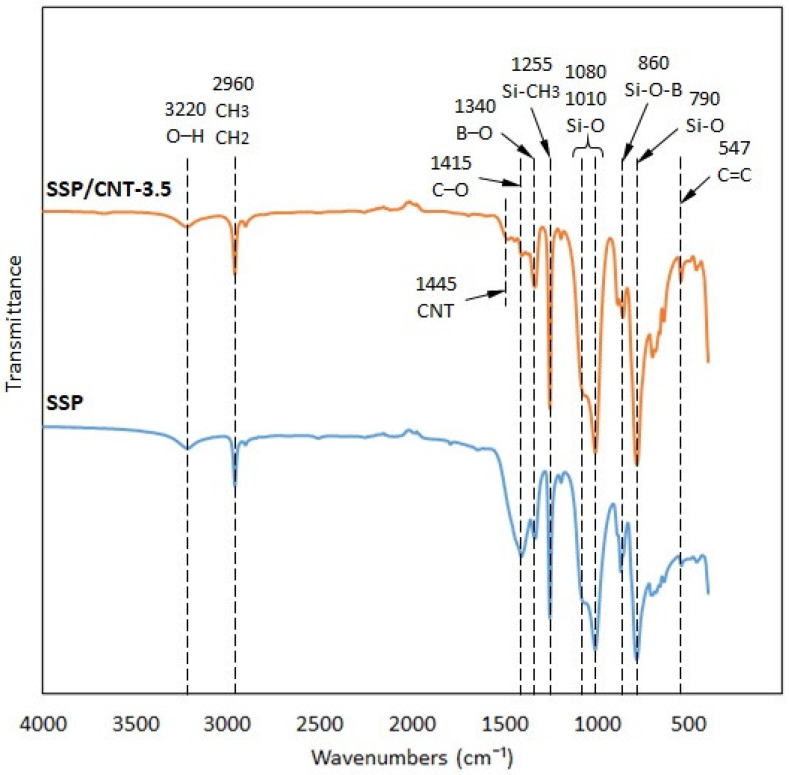
FTIR spectra of the specimens.

Figure 5 shows the FTIR spectra of the specimens. According to these results, the specimens show peaks at 3220 cm^−1^ (H-O), 2960 cm^−1^ (CH_3_ and CH_2_ groups), 1415 cm^−1^ (C-O), 1340 cm^−1^ (B-O), 1255 cm^−1^ (Si-CH_3_), 1080–1010 cm^−1^ (Si-O), 860 cm^−1^ (Si-O-B), 790 cm^−1^ (Si-O), and 547 cm^−1^ (C=C) [31,34,35]. The B-O, Si-O, and Si-O-B bonds are characteristic molecular chains for SSP, which correspond to the peaks at 1340 cm^−1^, 1080–1010 cm^−1^, and 860 cm^−1^, respectively. As discussed in the previous paragraph, Si-O bonds constitute the main molecular structure in the SSP. Due to the pyroboric acid source in the fabrication, B-O cross-bonds are also established in the material. These cross-bonds tie the Si-O long chains to each other, thereby providing stiffening characteristics for the polymer. Furthermore, a peak develops at 1445 cm^−1^ with the addition of CNT fillers to the polymer. The CNT inclusion in the polymer matrix leads to an increase in the relative intensity of the band at 547 cm^−1^, which is associated with the growing C=C groups.

**Figure 6:**
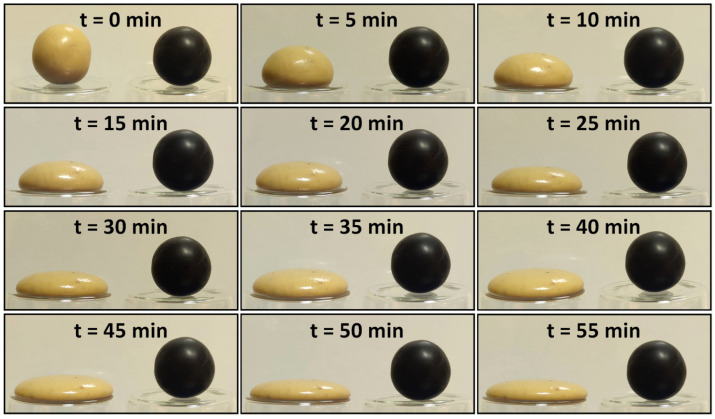
Shape stability of the pristine and 3.5 wt% CNT-included SSPs.

Figure 6 shows the images of the pristine and 3.5 wt% CNT-included SSPs in the shape stability tests. The test shows the cold flow behavior of the polymers, giving details about the long-term stability. As shown in the images, the pristine SSP has a gel-like texture that exhibits easy-to-flow properties. The material slightly flows by the gravitational effect and fully covers the pot within half an hour. It is possible to state that the pristine SSP has low shape stability for long-term durations. However, CNT reinforcement greatly enhances the shape stability of the material, thereby avoiding cold flowing in the SSP. It can be clearly seen in the images that the CNT-included SSP has almost no change during the 1 h resting period. The polymer matrix is reinforced with CNT networks due to the advanced mechanical properties and superior rigidity of the CNT fillers. It can be stated that the elastic part of the material is developed by the CNT fillers in the polymer matrix; therefore, shape stability is enhanced by hindering the cold flow phenomenon in the material. Recalling Figure 4, this is also compatible with the increasing trend in the storage modulus by including more CNT fillers in the SSP.

**Figure 7:**
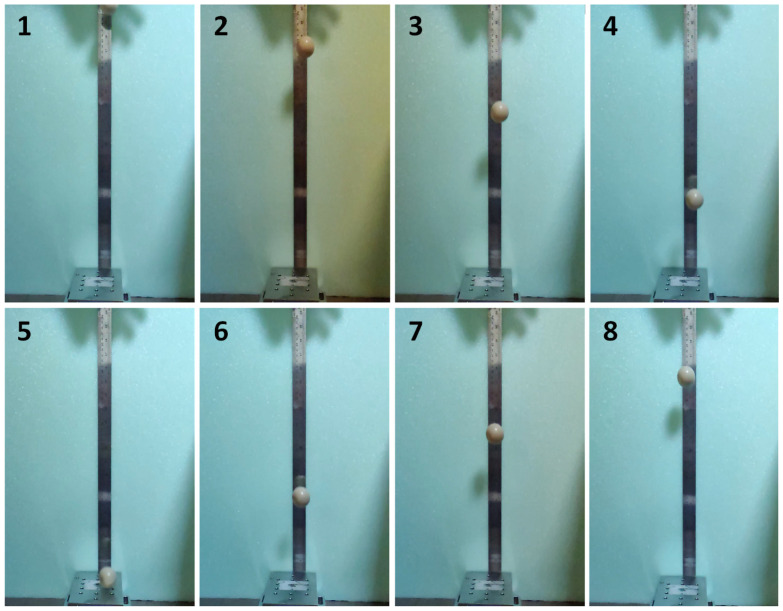
Instant images of the pristine SSP in the free-fall testing.

**Figure 8:**
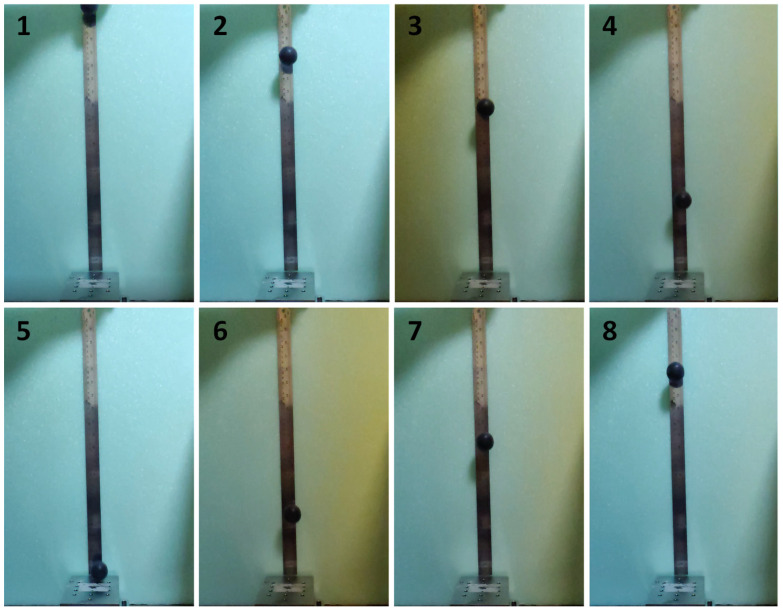
Instant images of the 3.5 wt% CNT-included SSP in the free-fall testing.

**Figure 9:**
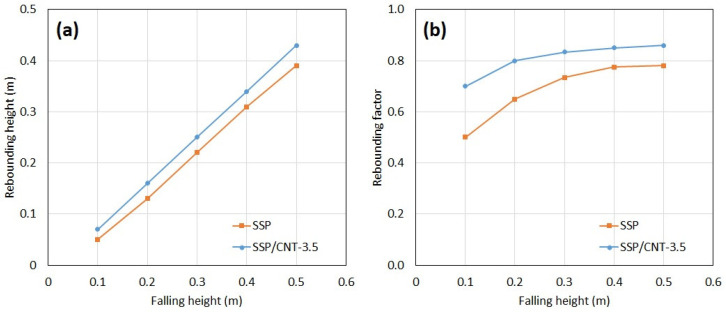
(**a**) Rebounding heights and (**b**) rebounding factors with respect to falling height.

Figure 7 and Figure 8 show instant images of the pristine and 3.5 wt% CNT-included SSPs in the free-fall tests, respectively. Frames 1 to 4 show the falling phase during the testing. The impact instant is shown in frame 5, whereas frames 6 to 8 show the rebounding phase in the testing. The peak point after rebounding is shown in frame 8. Figure 9 shows the rebounding heights and rebounding factors with respect to the falling height for the pristine and 3.5 wt% CNT-included SSPs. The rebounding factor is calculated by dividing the rebounding height by the falling height to evaluate the elastic behavior of the polymer balls. According to the results, the rebounding heights are higher for the CNT-included SSP. This is also reflected in the rebounding factors, which are higher with SSP/CNT-3.5 in comparison to SSP. This can be attributed to the growing elastic nature of the SSP due to the inclusion of CNT in the polymer matrix. As shown in the dynamic mechanical analysis results, CNT fillers enhance the elastic behavior of the material, thereby developing stiffer characteristics in the SSP. For this reason, the CNT-included SSP stores more energy during the impact process on the ground, releasing it just after ground contact, and consequently increasing the rebounding height in comparison to the pristine SSP. On the other hand, the rebounding factor shows a quick increase for falling heights from 0.1 to 0.3 m and then becomes almost a stable value for higher falling heights. This can be associated with the shearing rate during the impact process on the ground. Because the shearing rate is relatively small at lower falling heights, viscous behavior predominates over the material, thereby dissipating more energy than storing it. However, the material is subjected to higher shearing rates at higher falling heights; therefore, the elastic part of the material becomes stronger, consequently increasing the rebounding factor. These results are a good match with the dynamic mechanical analysis results that the storage modulus increases while drawing away from the loss modulus by increasing the frequency.

### 3.2. Electrical Properties

**Figure 10:**
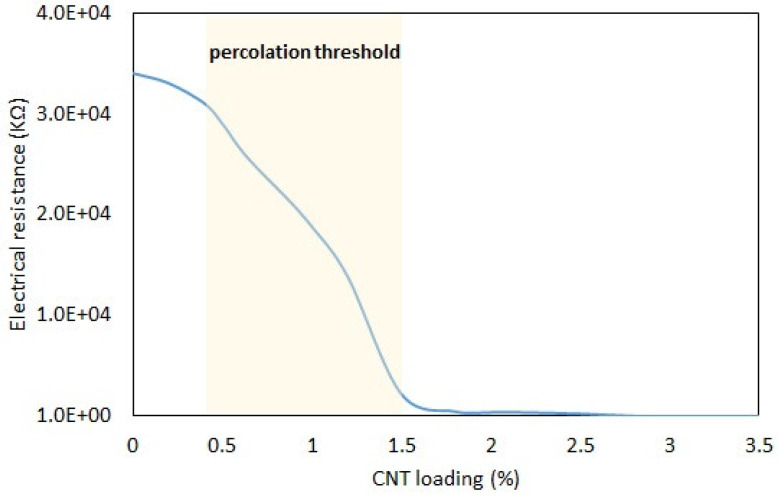
Electrical resistance vs. CNT content.

Figure 10 shows the electrical resistance with respect to the CNT concentration in the polymers. As shown in the chart, pristine SSP shows a significantly high electrical resistance that possesses fully dielectric properties. However, the material gradually becomes conductive in nature by including CNT fillers in the matrix. There is a critical filler amount, namely, the percolation threshold, at which the CNT fillers form conductive networks in the polymer. The percolation threshold is dependent on various factors, such as the morphology, size, and concentration of fillers, as well as the material properties in the matrix. The percolation threshold is found at CNT amounts between 0.5 and 1.5 wt%. Below the amount of 0.5 wt%, the polymer behaves as a dielectric material with drastically high electrical resistances. On the other hand, the material has significant conductive properties due to the effect of the CNT networks established in the microstructure beyond the loading of 1.5 wt%. According to the results, a constant resistance value is obtained at around 6 KΩ beyond the CNT loading of 2.5 wt%. Hence, good conductivity is achieved for the polymer. It is commonly known that CNT fillers are highly conductive materials that allow electron transport [36]. For this reason, CNT fillers are effective additives for gaining electrically conductive properties, as well as enhancing the mechanics of the materials. Based on the literature survey, similar results have been obtained in previous studies. Fan et al. [37] achieved a constant resistance of 13.5 KΩ beyond the CNT concentration of 2 wt%. Chen et al. [38] could lower the resistance value to 4 KΩ by using CNT fillers in the polymer matrix. These levels of electrical resistance are suitable for designing circuits. Intelligent applications such as wearable electronics can benefit from the SSP with CNT fillers of 2.5 wt% or more.

## 4. Conclusions

In this present study, an intelligent material, SSP, was investigated in terms of its mechanical and electrical properties. Despite its adaptive stiffening behavior, SSP is a dielectric material due to its polymeric nature. An attempt was made by integrating CNT fillers into this intelligent material to gain electrically conductive properties. According to the electrical resistance measurements, CNT fillers develop conductive paths inside the material, thereby gaining conductive behavior in this intelligent polymer. The percolation threshold is overtaken beyond the CNT loadings of 1.5 wt%, which means that conductive behavior is obtained at CNT concentrations higher than this limit. In addition to their contribution to the conductive properties, CNT fillers lead to stiffer behavior in SSP. CNT fillers enhance the elastic portion of the SSP due to their advanced strength and rigidity. Moreover, the crossover point for the SSP reduces to lower frequencies by including more CNT in the polymer matrix. This means that elastic behavior predominates over the material at lower loading rates as the CNT amount increases in the polymer. The CNT effect is clearly observed under dynamic conditions such as free-fall testing. Since the CNT content provides development in the elastic part of the material, the rebounding height increases with the CNT, including SSP. On the other hand, CNT additives avoid the cold flow phenomenon in the SSP by maintaining shape stability over a long period.

This study shows that CNT integration provides additional intelligent properties for SSP, such as gaining conductive behavior and tailoring mechanical properties by changing the filler amount in the polymer matrix. In recent years, SSP has been suggested for various applications, such as protective structures, vibration-damping systems, and wearable textiles. CNT inclusions have advantages in enhancing the intelligent behavior of SSP, as well as providing multi-functional roles from mechanical and electrical aspects. Despite the promising results and possible integration into the aforementioned applications, these intelligent materials should also be investigated under different conditions, such as variable temperature, moisture, and ultraviolet radiation. The mechanical and electrical stability of the SSP/CNT structures under various conditions will be studied in future works.

## Figures and Tables

**Table 1:** Design of the specimens.

Specimen	Content
SSP	Pristine SSP
SSP/CNT-0.5	0.5 wt% CNT in SSP
SSP/CNT-1	1 wt% CNT in SSP
SSP/CNT-1.5	1.5 wt% CNT in SSP
SSP/CNT-2	2 wt% CNT in SSP
SSP/CNT-2.5	2.5 wt% CNT in SSP
SSP/CNT-3	3 wt% CNT in SSP
SSP/CNT-3.5	3.5 wt% CNT in SSP

## Data Availability

The raw/processed data required to reproduce these findings cannot be shared at this time as the data also form part of an ongoing study.
